# Managing HCV Infection in Pediatric Age Group: Suggested Recommendations

**DOI:** 10.4103/1319-3767.65182

**Published:** 2010-07

**Authors:** Fazal A. Danish, Salman S. Koul, Fazal R. Subhani, Ahmed E. Rabbani, Saeeda Yasmin

**Affiliations:** Princess of Wales Hospital, Coity Road, Bridgend, United Kingdom CF31 1RQ; 1 Department of Medicine (Unit-I), Pakistan Institute of Medical Sciences (PIMS), Islamabad, Pakistan; 2 Department of Pediatrics, Holy Family Hospital, Rawalpindi, Pakistan; 3 Foundation University Medical College (FUMC), Rawalpindi, Pakistan; 4 Department of Surgery, Shifa Hospital, Islamabad, Pakistan

**Keywords:** Antiviral therapy, children, chronic hepatitis C

## Abstract

Hepatitis C virus (HCV) infection in children is different from the adult infection in many ways, like natural course of the disease; duration, therapeutic response and side effects profile of the drug therapy; and prognosis. Special considerations include consideration on what could be the appropriate time to investigate a suspected child, when to institute drug therapy and how to prevent vertical transmission. Although over the past one decade many landmark studies have greatly increased our insight on this subject, yet we are far from developing a consensus statement. In this article, a concise yet comprehensive review of HCV infection in children – diagnosis and treatment – is given, followed by suggested recommendations at the end. It is hoped that these recommendations will help develop local guidelines on this subject.

Antiviral therapy for chronic hepatitis C (HCV) has traditionally been contraindicated in children. A review of the recent literature, however, suggests that this view is no more valid. Many recent studies have suggested that antiviral therapy can be safely administered in children, with excellent results, though some important diagnostic and therapeutic considerations need to be addressed. In the light of current evidence, this article presents a practical and succinct diagnostic and therapeutic approach to treat HCV-infected children.

## DISCUSSION

Approximately, 0.2% and 0.4% of children under the age of 12 and between 12 and 19 years, respectively, are infected with hepatitis C in Pakistan;[1] Roughly, 75%-90% of them have HCV genotype 3. In USA, an estimated 240,000 children are infected with HCV, with viremia present in 50% to 75% of them.[2] The prevalence of HCV infection among women of childbearing age is estimated to be 1.2%, and the risk of HCV transmission at the time of delivery is 5%.[3] Whereas in the developed world maternal drug use and vertical (perinatal) transmission appear to be the major modes of HCV transmission in children,[4] in Pakistan we cannot overlook transmission by blood/ blood products transfusion. Thus all children who have ever been transfused unscreened blood/ blood products in Pakistan for any reason, and children born to infected mothers should be considered as potential cases of HCV/ HBV (hepatitis B virus) infection and investigated accordingly. In quite an appreciable percentage (63% in one study)[5] of HCV-positive pediatric age-group patients, no obvious cause of viral exposure is found.

The natural course of the disease is different in children as compared to adults. In children, HCV infection is more likely to be asymptomatic,[6] with normal or near-normal alanine aminotransferase (ALT) levels,[7] higher rates of spontaneous resolution (especially in genotype 3 cases)[8] and less probability of progression to end-stage liver disease.[9] Data however shows that the frequency of development of fibrosis is no different in children than in adults, though progression to cirrhosis is slower.[10–12] Interestingly, periportal fibrosis appears to be more common in children; it may worsen with age.[11,12] It appears that the probability of persistent viremia and development of end-stage liver disease is relatively higher in children who are infected with genotype la and also in those who have acquired disease via vertical route, from mothers who abuse drugs.[5] This probably represents the group of patients who should be offered the benefits of antiviral therapy early in the course of the disease. Conversely, spontaneous viral clearance is more likely with genotype 3 cases, as depicted in one regression analysis study by Cox (hazard ratio, 6.44; 95% confidence interval, 2.7-15.5).[5] In this group of patients, it is reasonable to give some time for spontaneous resolution to take place.

Diagnostic workup of children suspected of having chronic HCV should proceed similar to that of adults. A sensitive serological test (third-generation enzyme-linked immunoassay [EIA]) for the detection of anti-HCV antibodies followed by a sensitive HCV ribonucleic acid (RNA) assay for definite confirmation is the usual diagnostic protocol.[13,14] Unlike HBV cases, no test is currently available to determine infectivity in an HCV-infected patient. Therefore, *all* patients who are anti-HCV positive should be taken as potentially infectious. In anti-HCV–positive patients, a single positive HCV RNA assay confirms the diagnosis. Since HCV RNA is normally present only intermittently in the circulation, a single negative assay does not rule out the diagnosis of HCV infection. Thus all negative test results should be reconfirmed with a repeat test done approximately one to two months later so as to reliably rule out the presence of ongoing HCV infection. Improper collection, handling and storage of blood samples are the major causes of false-negative HCV RNA assay results. The special precautions that need to be observed in all cases include urgent shipping of the samples on dry ice; rapid separation of the serum within two to four hours of collection; and storage at –20°C.

Because of the probability of spontaneous clearance of HCV RNA from circulation during the first few years of life, infants born to HCV-infected mothers (particularly, genotype ‘2 and 3’ cases, which represent most of the Pakistani patients) should best be given time for spontaneous resolution to take place. Investigative workup can thus be deferred till 18 months of age or later. Since antiviral treatment is widely considered as contraindicated in children under the age of 3 years due to potential neurotoxicity, there is no need to induce undue anxiety in the family just to have an earlier diagnosis. However, if parents insist on an earlier diagnosis, a qualitative polymerase chain reaction (PCR) test for HCV RNA may be performed at or after the infant’s first healthy-child visit at 1 to 2 months of age. Anti-HCV antibodies passively acquired by infants from the infected mothers may persist in the circulation for up to 12 months. Therefore, serological testing (anti-HCV detection by enzyme immunoassay) should better be avoided in infants born to infected mothers. Because of the possibility of contamination with the maternal blood, cord blood should never be used for any HCV-related testing.[15]

The role and utility of liver biopsy in hepatitis C cases is still debatable, and no definite consensus exists in any guideline. One subgroup of patients in whom liver biopsy is most likely to be useful is those HCV-positive patients in whom aminotransferase levels remain persistently normal.[16] As is well known that ALT levels do not correspond accurately to the degree of hepatic fibrosis, in patients with persistently normal aminotransferase levels, the only way to reliably determine the extent and severity of liver disease and thus to make timely decisions regarding therapeutic interventions is to go for liver biopsy. If liver biopsy in such patients shows only a minimal fibrosis limited to the portal tract (Metavir[17] score, <2; or Ishak[18] score, < 3), initiation of interferon therapy may be delayed/ individualized. Repeat biopsies done at intervals of four to five years (or may be more frequently) can be used to monitor the progression of fibrosis in such cases. If repeat biopsies show worsening of fibrosis, especially more-than-portal fibrosis (i.e. Metavir score, ≥2; or Ishak score, ≥3), patients should be offered antiviral therapy lest fibrosis worsens and cirrhosis develops, when the treatment success rate and prognosis will be relatively poorer.[19]

It is suggested that all ≥3 years old patients with positive serology and HCV RNA be offered antiviral therapy. Children with persistently normal ALT levels, however, can be monitored with serial liver biopsies as mentioned above.

Controversy exists regarding who to treat and who not to treat amongst infected children. Since disease progression is less likely in children, it appears reasonable not to expose children to the adverse events associated with antiviral therapy. On the other hand, when we look at the life expectancy of an average child, it does not seem rational to let a child live for 50 or more years with ongoing infection. It is pertinent to mention here that although reported in the literature,[20] the lifetime risk of development of end-stage liver disease and hepatocellular carcinoma (HCC) in HCV-infected children is very low.

Antiviral therapy should not be given in children less than 3 years of age.[13,21] The reason is potential neurotoxicity of interferon and thus its deleterious effects on the developing brain.[13] Because of the high rate of spontaneous resolution, the need for interferon therapy, if any, is only minimal in children less than 3 years of age. Children aged 3 to 17 years who are infected with hepatitis C and are considered appropriate candidates for treatment may be offered antiviral therapy.[13,21,22] The recommended dose regimen is interferon alfa-2b (3 MU/m^2^ subcutaneously thrice weekly) and ribavirin (15 mg/kg orally) for 24 and 48 weeks in genotypes ‘2 and 3’ and 1 cases, respectively.[13,14,21] Children should be treated by only those who have experience in dealing with patients of pediatric age group.

Although more validating studies are needed, recently pegylated interferon (PEG-IFN)-alpha2b and ribavirin (RBV) combination therapy is being tried in children, with reasonably good results. In one study,[23] 30 children between 3 and 6 years of age were selected for antiviral therapy based on positive HCV RNA for ≥3 years and elevated ALT levels. They received PEG-IFN-alpha2b 1.0 *μ*g/kg/wk plus ribavirin 15 mg/kg/d for 24 weeks (genotype 2/3) or 48 weeks (genotype 1/4). Primary endpoint, i.e., attainment of sustained virologic response (SVR) defined as undetectable HCV RNA [< 50 IU/mL] at week 24 of follow-up, was achieved in 50% of the patients (3/3 in genotype 3 cases; 12/27 in genotype 1/4 cases). No patient required ribavirin dose reduction; because of neutropenia, PEG-IFN-alpha2b dose was reduced in 23% of the patients and stopped in 3 subjects.

A recent comparative analysis of efficacies of different therapeutic options available for children has revealed that PEG-IFN-alpha 2b-ribavirin combination therapy yields better results (in terms of % ETR [end-of-treatment response] and SVR achieved)[24] as compared to non-pegylated interferon monotherapy or its combination with ribavirin.

With the above-mentioned standard therapy, compared to adults, higher SVR rates (genotype 2/3 [84%]; genotype 1 [36%])[25,26] and fewer adverse events have been reported in children. The higher SVR rate observed in children might be the result of the earlier stage of the disease, higher relative IFN dosage and lack of comorbid conditions.[24] It is not clear at this point whether to use early virologic response (EVR) as a criterion, similar to adults, to stop therapy at week 12 or not. Refer to Table 1 for definitions of treatment responses, and Table 2 for a schematic layout of the management plan in children with genotypes ‘2 and 3’ — the most prevalent genotypes in Pakistan.

**Table 1:** Definitions of treatment responses

Rapid virologic response (RVR)	Qualitative HCV RNA assay done at 4 weeks comes out to be negative (<50 lU/mL)
Early virologic response (EVR)	Quantitative HCV RNA assay done at 12 weeks:
	Comes out to be negative — called early virologic clearance (EVC) or aviremic response
	Shows a decline in the HCV RNA titer (compared with the pretreatment assay) of ≥ 2 log — called partial virologic response (PVR) or viremic response
Nonresponders	Quantitative HCV RNA assay done at 12 weeks showing either no decline in the HCV RNA titer (compared with the pretreatment assay) or a decline of < 2 log
End-of-treatment response (ETR)	Qualitative HCV RNA assay done on completion of the recommended duration of the course comes out to be negative
Sustained virologic response (SVR)*	Qualitative HCV RNA assay done 24 weeks after completion of the recommended duration of the course comes out to be negative
Relapsers	Qualitative HCV RNA assay done on completion of the recommended duration of the course is negative; but 24 weeks later, the assay done to confirm SVR comes out to be positive

* Achievement of SVR is generally considered as the marker of eradication of HCV infection. Almost all such patients show EVC or PVR on 12-week assay

**Table 2:** Suggested management plan in children with genotypes '2 and 3'

HCV RNA Assay	Recommendation as per the HCV RNA assay result
Week 4 qualitative HCV RNA assay*	
Negative assay (<50 lU/mL), i.e., a case of RVR	Institute a standard treatment course of 24 weeks. Although, a few studies have shown attainment of comparable SVR rates in this subgroup with shortened treatment courses of 12-16 weeks, more data is needed to validate this recommendation in pediatric age group
Positive assay	Give treatment for the standard duration of 24 weeks† (may be 36-48 weeks)
Week 24 qualitative HCV RNA assay	
Negative assay, i.e., a case of ETR	Successful therapy. Needs a repeat qualitative HCV RNA assay at week 48 (24 weeks after ETR) to establish SVR
Positive assay	Treatment failed
Week 48 qualitative HCV RNA assay	
Negative assay, i.e., a case of SVR	HCV infection got eradicated

* The newly recommended week-4 qualitative HCV RNA assay helps modify the duration of the therapy based on viral kinetics. On one hand, this approach helps maximize the SVR rates and on the other hand, limits the toxicities and cost associated with the extended treatment courses. Achievement of RVR means that we can consider shortening the treatment course

† SVR rates achieved in this subgroup are relatively poor. Thus prolonged therapy (>24 weeks) may be considered in this subgroup, although more evidence is needed at this time for a definite recommendation

Nonresponders are those patients in whom the quantitative HCV RNA assay done at 12 weeks into the therapy shows either no decline in the HCV RNA titer (compared with the pretreatment assay) or a decline of < 2 log.[13] Relapsers, on the other hand, are those patients in whom the qualitative HCV RNA assay done at the end of the treatment course comes out to be negative (<50 IU/mL), i.e., end-of-treatment response achieved; but 24 weeks later, qualitative HCV RNA assay done to ascertain sustained virologic response comes out to be positive.[13] In Pakistani patients, since genotypes 2/3 (the most treatment-responsive genotypes) accounts for almost 75% to 90% of all cases, we do not recommend repeat PCR testing at week 12 in our patients.

How do we approach nonresponders and relapsers basically depends upon the previous drug regimen administered in the patients (peginterferon-ribavirin combination; nonpegylated interferon–ribavirin combination; peginterferon monotherapy; nonpegylated interferon monotherapy) and the presence of negative predictors to drug therapy. Patients who were prescribed any regimen other than peginterferon-ribavirin combination therapy can be prescribed this regimen regardless of the genotype[13]; and sustained virologic response rates of 25% to 40% for nonpegylated interferon monotherapy cases and 10% for nonpegylated interferon–ribavirin combination therapy cases can be expected.[26]

Antiviral therapy needs to be monitored to look for the development of potentially serious side effects and also to determine the response to therapy [Table 3]. Monitoring of antiviral therapy includes checking complete blood counts (CBC) at weeks 1, 2, 4, 6, 8 and then monthly; every three months, all baseline investigations should be repeated, including liver function tests (LFT’s), creatinine, glucose and thyroid function tests (TFT’s).

**Table 3:** Monitoring of antiviral therapy

Fortnightly:	CBC at weeks 1, 2, 4, 6, 8 and then monthly
Week 4:	Qualitative HCV RNA assay at week 4 in both genotype 1 and ‘2 and 3’ cases to assess for RVR
Week 12:	Qualitative HCV RNA assay at week 12 in genotype 2 or 3 cases only to assess for EVR
Every 3 months:	LFTs, creatinine, glucose and TSH
Week 24:	Qualitative HCV RNA assay at week 24 in only those genotype 1 cases wherein EVR is attained at week 12
Week 48:	Qualitative HCV RNA assay at week 48 in genotype ‘2 and 3’ cases to determine SVR
	Qualitative HCV RNA assay at week 48 in genotype 1 cases to determine ETR
Week 72:	Qualitative HCV RNA assay at week 72 in genotype 1 cases to determine SVR

Unlike adults, children surprisingly appear to tolerate interferon therapy much better. With the exception of transient growth inhibition that appears to reverse following termination of interferon therapy, no serious side effects have been reported with interferon use in different studies.[27,28] As with ribavirin therapy, the incidence of hemolytic anemia appears to be less than that in adults;[29] also, it appears that the incidence does not rise when higher doses (15 mg/kg) are used as compared to lower doses (8-12 mg/kg).[22] Anemia, however, is a particular problem in those having renal insufficiency, cirrhosis, thalassemia or HIV co-infection. There are reports of ribavirin-induced worsening of anemia in such patients with consequent rise in transfusion requirements (especially, in thalassemia patients).[3] Anemia usually develops within the first four weeks of starting antiviral therapy and persists till the end of the course.[30] Almost 40% of patients suffer a drop in Hb concentration of ≥3 g/dL.[30] Most published studies recommend RBV dose reduction if Hb level falls to or below 10 g/dL, and discontinue it if it falls to <8 g/dL.[21,31] The current recommendation is to reduce IFN dose if neutrophil count falls to < 0.5 × 10^9^ /L, and discontinue it if it falls to <0.3 × 10^9^ /L.[13,31] Regarding platelet count, it is recommended to reduce IFN dose if platelet count falls to < 30 × 10^9^ /L, and discontinue if it falls to <20 × 10^9^ /L.[21,31] Because of the risk of development of life-threatening infections, patients who already have neutropenia or thrombocytopenia below the permissible limits (neutrophil count >1500/mm^3^ and thrombocyte count >75,000/mm^3^ ) should not be started on antiviral therapy. Although hematopoietic growth factors (erythropoietin and filgrastim) have been used in adults to help avoid antiviral dose reductions and attain *optimum adherence* (defined as the administration of interferon-ribavirin combination therapy in an optimum dose for more than 80% of the prescribed duration),[32] our experience with these drugs in children is almost nonexistent. Therefore, in spite of promising results in adults, the use of these agents as adjuncts to antiviral therapy is not recommended at this moment.

Traditionally, despite the known theoretical benefits of antiviral therapy (improvement in liver histology, partial reversal of established cirrhosis and prevention of life-threatening complications), many cirrhotic patients have not been offered antiviral therapy. Current literature review, however, shows that because of the unstandardized dosage schedules being administered over variable periods of time in the past studies, we have under/over estimated the potential benefits and risks of antiviral therapy in decompensated cirrhotic patients. Based on the current literature review, it is suggested that cirrhotic patients with a CTP score ≤9 and a decompensated event that abated with common management may be considered for antiviral therapy,[33,34] although more data in pediatric age group is needed to recommend routine usage of this therapy.

It is suggested that all pregnant ladies from countries with high seroprevalence undergo routine testing for HCV. Since vertical transmission usually occurs at the time of birth, in order to reduce its risk, most pediatricians recommend going for delivery within 6 hours of membrane rupture in HCV-infected mothers and avoidance of the use of fetal scalp monitors. No measure, however, fully prevents vertical transmission.[35,36] Current evidence has not proved cesarean section to be a particularly useful way of reducing the transmission risk. Thus most authorities in this field do not recommend the routine use of this procedure. Also, breastfeeding by HCV-infected mothers is permissible as there is little evidence that HCV is transmitted in breast milk unless nipples are cracked or bleeding. Horizontal transmission from child to child is also a rarity, and thus HCV-infected children can intermingle with other children as normal.

## CONCLUSION

A large-scale, population-based seroprevalence survey is the need of the hour in order to reliably estimate the true disease burden of hepatitis C in Pakistan. The study design should take into account the risk factors particularly strong in our population (e.g., unscreened blood transfusion). A well-streamlined surveillance, data collection and reporting process should also be developed at national level, in order to determine the true incidence of new cases. Diagnostic workup (serology by third-generation EIA/ ELIZA followed by qualitative HCV RNA assay) of children suspected of having chronic HCV should proceed similar to that of adults. Liver biopsy may be considered in HCV-positive children with persistently normal aminotransferase levels. Because of the potential interferon-induced neurotoxicity, antiviral therapy is generally considered contraindicated in children less than 3 years of age. Infected children aged 3 to 17 years who are selected for treatment may receive therapy with interferon alfa-2b (3 MU/m^2^ three times a week) and ribavirin (15 mg/kg/d) for 24 and 48 weeks in genotypes ‘2 and 3’ and 1, respectively. Some recent studies have shown that pegylated interferon-ribavirin combination therapy yields better results in terms of attainment of ETR and SVR rates than non-pegylated interferon. In nonresponding patients/ relapsers, peginterferon-ribavirin combination therapy may be prescribed regardless of the genotype, provided the same was not given beforehand. Cirrhotic patients with a CTP score ≤9 and a decompensated event that abated with common management, may be considered for antiviral therapy, although more data in pediatric age group is needed to recommend routine usage of this therapy. Despite promising results in adults, the use of hematopoietic growth factors (erythropoietin and filgrastim) as adjuncts in the management of HVC infection in chidren, is not recommended at this moment.
